# Response to Induction Therapy in Pediatric Hodgkin’s Lymphoma: Performance of First-Order Texture Parameters of CT Images

**DOI:** 10.5334/jbsr.2752

**Published:** 2022-05-10

**Authors:** Margaux Doillon, Carole Durot, Claire Pluchart, Claude Marcus, Manel Djelouah, Aline Carsin-Vu

**Affiliations:** 1 Department of Radiology, American Memorial Hospital, 47 rue Cognacq-jay, FR; 2 Department of Oncopediatrics, American Memorial Hospital, 47 rue Cognacq-jay, FR; 3 Department of Radiology, American Memorial Hospital, 47 rue Cognac q-jay, FR

**Keywords:** Hodgkin lymphoma, Pediatric, Computed tomography, Computer-assisted image analysis, Therapeutics

## Abstract

**Objectives:**

The aim of this study was to examine whether texture analysis features on pretreatment contrast-enhanced CT images could predict adequate response (AR) or inadequate response (IR) after two cycles of chemotherapy in pediatric Hodgkin’s lymphoma (PHL).

**Materials and methods:**

This retrospective single-center study included 32 children and adolescents with HL. Texture analysis was independently performed by two radiologists using pretreatment CT scans. The mean gray level, standard deviation, entropy, kurtosis, and skewness were derived from pixel distribution histograms before and after spatial filtration ranging from two (fine texture) to six (coarse texture). Interobserver reliability was studied using interobserver correlation coefficients (ICCs) to select texture parameters. Relationships between early response assessment (ERA) to induction therapy and associated factors were studied using Student’s *t*-tests and a lasso penalized logistic regression analysis.

**Results:**

Of the 32 patients, IR was observed in 13 and AR in 19. Inter-reader agreement was good to excellent (ICC > 0.75) for all parameters except skewness and kurtosis without filtration and at spatial scale filtration (SSF) = 2. These parameters were excluded from the analysis. The *t*-test identified only entropy at SSF = 2 (*p* value = 0.039) as a potential predictor of ERA. No parameters were significantly associated with ERA, according to a lasso penalized logistic regression.

**Conclusion:**

No textural parameters were identified as predictors of ERA after two cycles of chemotherapy in PHL.

## Introduction

Pediatric Hodgkin’s lymphoma (PHL) constitutes approximately 40% of all childhood lymphomas and is the most common malignancy in adolescents and young adults [1]. It accounts for approximately 7% of pediatric malignancies and 1% of childhood cancer-related deaths in the United States [2]. PHL prognosis is good, and up to 80% of PHL patients treated with the current radio/chemotherapy protocols are cured [1]. Treatment intensity within standardized treatment protocols is based on different treatment groups (TGs) defined by EuroNet-PHL-C1 [3,4].

Unfortunately, a significant number of PHL patients suffer from treatment-related morbidity and mortality caused by chemotherapy and radiotherapy. The good prognosis of Hodgkin’s disease is often accompanied by elevated risks of second primary cancers, cardiovascular disease, and infections. In several studies, mortality resulting from secondary cancers and heart disease exceeded lymphoma-related deaths after 15 to 20 years of follow-up [1,5,6]. For these reasons, reducing treatment-associated toxicity while maintaining high cure rates has become a major issue. In patients with adequate response (AR), radiotherapy can be omitted after two cycles of chemotherapy, irrespective of the TG. In these cases, the late effects of radiotherapy, especially secondary malignancies, can be avoided [4]. Furthermore, an initial prediction of response to induction therapy could even allow individualized treatment intensification to prevent inadequate response (IR) a priori.

Texture analysis, which provides an objective, quantitative assessment of tumor heterogeneity by analyzing the distribution and relationship of pixel or voxel gray levels in an image [7], could be an useful method to individualize chemotherapy. In various types of cancers, tumor texture analysis has been reported as a possible tool for assessing survival [8,9,10,11,12]. Studies in adult patients with HL have suggested that texture analysis of CT images could provide prognostic information complementary to 18-FDG PET [13,14,15]. In children with HL, no studies have been done on the prognostic role of CT texture analysis. However, a feature of texture parameters is their potential lack of inter-reader reproducibility [16].

The purpose of this study was to determine whether texture analysis features on pretreatment contrast-enhanced CT images could predict AR or IR after two cycles of chemotherapy in children and adolescents with HL and serve as an additional prognostic factor allowing treatment adaptation. A secondary end point was to assess inter-reader reproducibility of texture features beforehand according the texture parameters and filter coarseness.

## Materials and Methods

### Study population

This retrospective study included all pediatric patients with classical PHL who were treated according to EuroNet-PHL-C1 guidelines between 2010 and 2019 at *institute*. All patients had a histological diagnosis of classical HL performed on lymphoid tissue from an excisional biopsy. Exclusion criteria included the following: age older than 18 years, previous chemotherapy, and images with artifacts or insufficient resolution. All patients underwent an initial CT scan and an initial PET-CT. They were divided into three TGs according to the EuroNet-PHL-C1 reference stages, depending on the classical Ann Arbor staging system, the elevated erythrocyte sedimentation rate, and tumor bulk [3,4]. The different groups received the corresponding treatment schedules.

In accordance with French law, this retrospective study of medical records was authorized by the Commission Nationale de l’Informatique et des Libertés (authorization number 1118523), allowing the computerized management of medical data at *institute*. The participants were informed of the possibility that their information would be used for biomedical research purposes, and they had the right to decline.

### CT examination

All patients underwent a contrast-enhanced CT scan covering the cervical region, the chest, abdomen, and pelvis. Most of the scans were performed at the *institute* (n = 26) using one of the following multi-detector CT scanners: Discovery HD 750, GE Healthcare (n = 23) or GE Revolution Evo, GE Healthcare (n = 3). Four CT examinations were provided by small nearby centers (one from *city*, GE Revolution Evo; two from *city*, Siemens Definition 128; and one from *city*, Siemens Somatom Go All). Two CT scans were performed in private facilities in *city* (Siemens Power Scope 32).

A volume of 2 mL/kg of body weight of non-ionic contrast material (Xenetix 350; Iomeron 350) was injected into an antecubital vein. Chest images were obtained at an arterial phase 35 s after contrast material administration, and abdominal and pelvic images were obtained at a portal-venous phase (70 s).

### CT texture analysis

CT texture analysis was performed on the pretreatment CT examination using the commercially available TexRAD software (TexRAD Ltd). Two radiologists (R1 and R2, with seven years and four years of experience, respectively, in oncological imaging) selected together two or three target lesions for each patient. The lesions corresponded to either pathologic lymph nodes (in most cases), the thymus, or to an extra-nodal lesion for stage IV.

**Figure 1:**
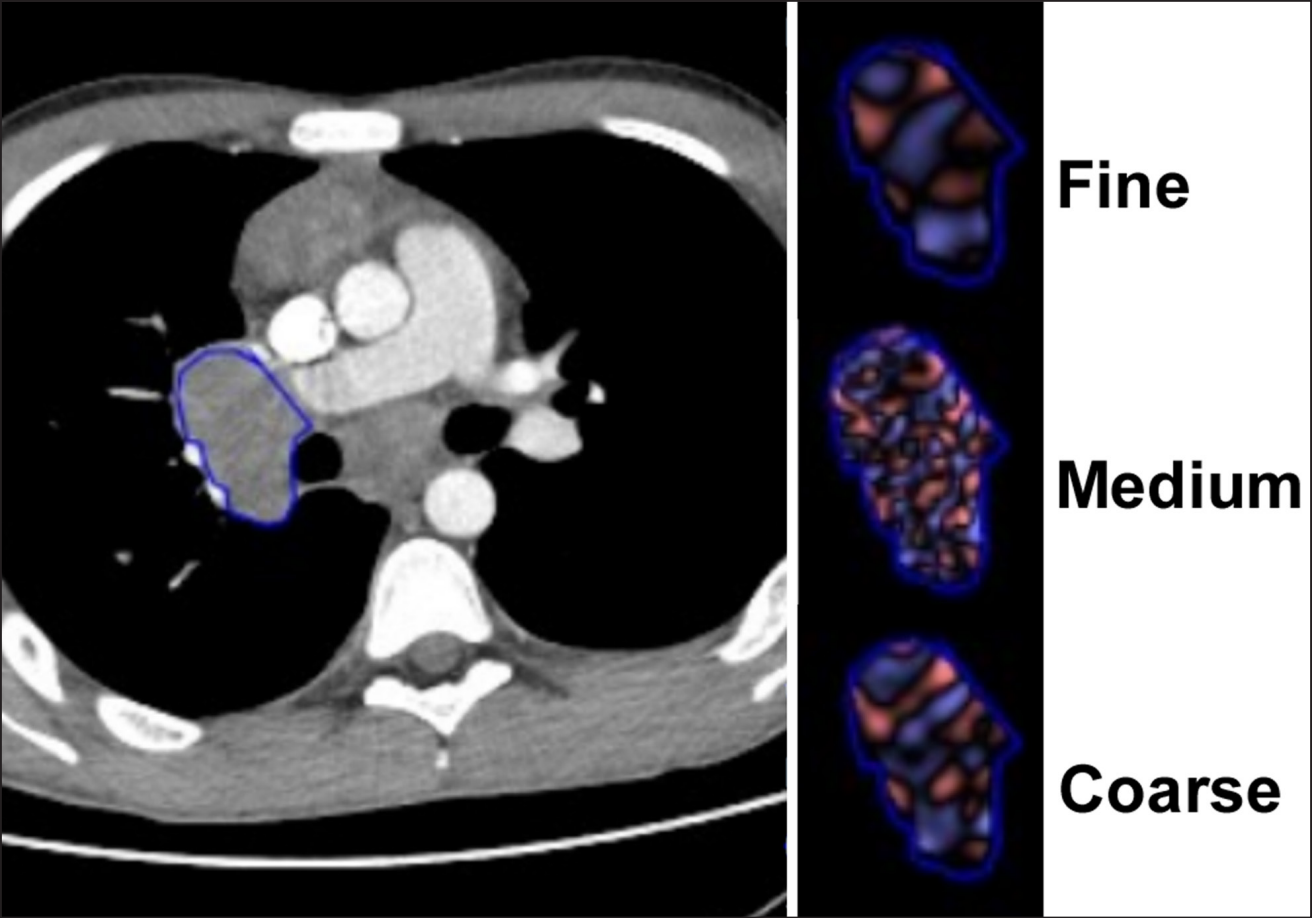
CT images in 17-year-old young men with Hodgkin’s lymphoma stage IIB. Illustration of nodal lesion delimitation, image filtration.

**Figure 2:**
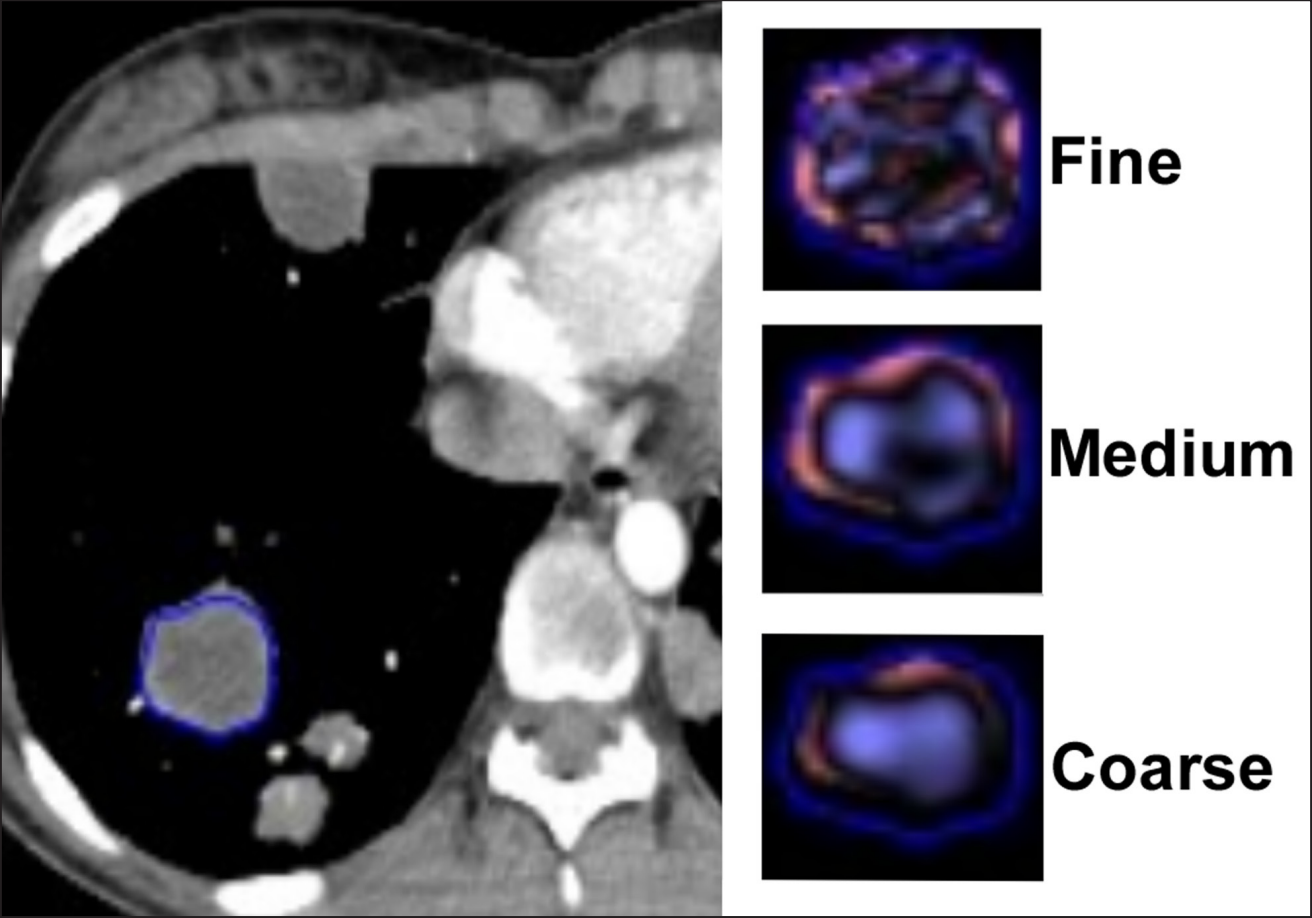
CT images in 15-year-old young women with Hodgkin’s lymphoma stage IV. Illustration of the pulmonary lesion delimitation, image filtration.

Afterwards, both radiologists independently placed a free-hand region of interest (ROI) encompassing each entire lesion (Figures 1 and 2). CT texture analysis was performed in a two-step process that included image filtration and histogram quantification steps. Spatial scale image filtration (SSF) selectively extracted features with different texture, corresponding to fine (SSF = 2, object radius of 2 mm), medium (SSF = 3–5, object radius of 3–5 mm), and coarse (SSF = 6, object radius of 6 mm) scales, using a Laplacian of Gaussian spatial band-pass filter. Quantification of the histogram distribution within the ROI allowed the extraction of the following five first-order histogram parameters: mean gray-level intensity (mean), standard deviation (SD), entropy (irregularity), kurtosis (peakedness/flatness of the pixel histogram), and skewness (asymmetry). For each patient, the mean value of each texture parameter among the lesions was calculated. After the inter-reader agreement, was evaluated, the values of the two observers were averaged for statistical analysis.

### Early response assessment (ERA)

ERA was performed according to the EuroNet-PHL-C1 after two cycles of induction chemotherapy based on a PET-CT and a CT. Patients were then classified into two response groups (AR or IR), according to the EuroNet-PHL-C1 reference stages [3,4]. The primary endpoint was binary: AR or IR after induction treatment.

### Statistical analysis

The inter-observer agreements for the measurements of the texture parameters were assessed using intraclass correlation coefficients (ICCs) for each pair of variables. An ICC was considered poor if it was below 0.50, moderate between 0.50 and 0.75, good between 0.75 and 0.90, and excellent above 0.90. The reliability was considered satisfactory for ICCs ≥ 0.75 [17].

After determining that the variables were normally distributed, we used Student’s *t*-test to study the association between ERA and the CT texture parameters with an inter-reader agreement greater than 0.75. A multivariate analysis was performed to identify independent predictors of AR and IR among clinical and texture parameters. To take into account the correlation between the estimates of each texture parameter and the different filter values, as well as the small number of events compared with the number of included covariates, a multivariate L1 (least absolute shrinkage and selection operator [Lasso]) penalized logistic regression model was built to select texture parameters. The lasso method allows variable selection by shrinking coefficient weights to zero for variables unrelated to outcome.

Statistical analyses were performed using RStudio Desktop (version 1.4.1717, RStudio, PBC), and *p* values < 0.05 were considered statistically significant.

## Results

### Patient characteristics

The study population comprised 32 patients. IR was observed in 13 of the 32 patients. A total of 78 lesions were analyzed: 79.5% from lymph nodes, 15.5% from the thymus, and 5.1% from an extra-nodal organ (lungs). The patients’ clinical and lesion characteristics are reported in Table 1.

**Table 1:** Patient characteristics.


PARAMETER	ALL PATIENTS (%)	PATIENTS WITH AR (%)	PATIENTS WITH IR (%)

TOTAL	32	19	13

Sex			

Male	15 (46.9%)	9 (47.4%)	6 (46.2%)

Female	17 (53.1%)	10 (52.6%)	7 (53.8%)

Stage			

I	2 (6.25%)	2 (10.5%)	0

II	10 (31.25%)	6 (31.6%)	4 (30.8%)

III	10 (31.25%)	6 (31.6%)	4 (30.8%)

IV	10 (31.25%)	5 (26.3%)	5 (38.4%)

TG			

1	4 (12.5%)	4 (21.1%)	0

2	13 (40.6%)	7 (36.8%)	6 (46.2%)

3	15 (46.9%)	8 (42.1%)	7 (53.8%)

Lesions			

Lymph node	62 (79.5%)	35 (77.8%)	27 (81.8%)

Thymus	12 (15.4%)	8 (17.8%)	4 (12.1%)

Lung	4 (5.1%)	2 (4.4%)	2 (6.1%)


### Inter-reader agreement

Table 2 displays the results for interobserver agreement with different filtration. Inter-reader agreement was acceptable (ICC > 0.75) for all parameters excepted skewness without filtration (ICC = 0.637), skewness at SSF = 2 (ICC = 0.666), kurtosis without filtration (ICC = 0.588), and kurtosis at SSF = 2 (ICC = 0.491). These last four parameters were excluded from the analysis because they were not reproducible.

**Table 2:** Inter-reader agreement.


PARAMETER ICC (INTERVAL)	SSF0	SSF2	SSF3	SSF4	SSF5	SSF6

Mean	0.995 (0.989–0.998)	0.791 (0.615–0.892)	0.773 (0.52–0.86)	0.837 (0.694–0.917)	0.837 (0.695–0.917)	0.849 (0.714–0.923)

Standard deviation	0.884 (0.752–0.945)	0.965 (0.93–0.983)	0.974 (0.947–0.987)	0.946 (0.892–0.973)	0.923 (0.85–0.962)	0.924 (0.851–0.962)

Entropy	0.96 (0.87–0.984)	0.942 (0.883–0.971)	0.896 (0.799–0.947)	0.898 (0.82–0.948)	0.889 (0.786–0.944)	0.827 (0.677–0.911)

Mean of positive pixels	0.997 (0.994–0.999)	0.97 (0.939–0.985)	0.953 (0.906–0.977)	0.981 (0.789–0.945)	0.824 (0.672–0.91)	0.804 (0.639–0.899)

Skewness	**0.637** (0.376–0.805)	**0.666** (0.42–0.821)	0.832 (0.675–0.916)	0.901 (0.801–0.951)	0.84 (0.697–0.918)	0.894 (0.794–0.947)

Kurtosis	**0.588** (0.31–0.774)	**0.491** (0.172–0.716)	0.893 (0.795–0.946)	0.863 (0.74–0.93)	0.894 (0.794–0.947)	0.89 (0.714–0.923)


The bottom ICCs (< 0,75) are in bold. ICC = Intraclass Correlation Coefficient.

### CT texture parameters and early response assessment (ERA)

The results of the univariate analysis are presented in Table 3. The *t*-test identified one texture parameter as a potential predictor of ERA: entropy at a fine scale (SSF = 2), *t* = 2.16 [CI 95%], *p* value = 0.039. The other parameters were not significant (*p* ≥ 0.05). The lasso penalized logistic regression analysis did not identify texture parameters as potential predictors of ERA.

**Table 3:** Univariate analysis results.


PARAMETER P-VALUE	SSF0	SSF2	SSF3	SSF4	SSF5	SSF6

Mean	0.093	0.34	0.14	0.30	0.36	0.33

Sd	0.11	0.05	0.27	0.39	0.79	0.93

Entropy	0.10	0.04*	0.31	0.46	0.97	0.79

Mpp	0.10	0.15	0.46	0.66	0.99	0.89

Skewness			0.19	0.56	0.62	0.78

Kurtosis			0.77	0.90	0.67	0.39


* P < 0.05, indicating a significant difference.

## Discussion

In previous studies, CT texture analysis was able to predict overall survival in many types of cancer and response to treatment [8,9,10,11,12]. CT texture analysis has recently emerged as a new technique allowing quantitative analysis of tumor heterogeneity, which is an approach to assess tumor hypoxia and angiogenesis [7]. To our knowledge, our study is the first to attempt predicting ERA with CT texture analysis at baseline.

We manually drew the tumor segmentation, which could have induced a subjective tendency or bias, as CT texture analysis is sensitive to intra- and interobserver manual segmentation [16]. To assess the interobserver variability in our study, a second radiologist re-segmented all images independently. In similar studies, inter-reader reproducibility was satisfactory [10,12] and it improves results to systematically check the reproducibility before the evaluation of radiomic analysis [16]. In PHL, we showed a good to excellent inter-reader reproducibility of CT texture analysis for all parameters except skewness and kurtosis without filtration or with weak filtration (SSF = 2). Previous texture studies showed similarly low reproducibility when the original images were unfiltered [16]. This factor may lead to analyzing images only with a high filtration so that reliable parameters are used, possibly decreasing the number of observed parameters, which is useful for large series and clinical practice.

We took multiple texture parameters into account by doing a lasso analysis to try to identify the parameters significantly related to ERA. No significant link was found between the texture parameters and ERA, but another element which could be considered is the evolution of tumor heterogeneity during treatment. Reinert et al. [15] found significant modification of texture parameters between baseline and interim imaging evaluation and between partial and complete remission on interim imaging (PET or CT) in an adult HL population.

Our study had several limitations, which can partly explain the lack of meaningful texture criteria. First, our study was exploratory and included few patients. More comprehensive investigations of texture parameters in PHL and their evolution during treatment are needed in larger populations. These types of studies would allow for selecting the parameters related with good outcome. The choice of target lesions can also be discussed. We chose to include nodal and extra-nodal lesions, as in clinical practice, but this factor could have affected the texture parameters after averaging because of the histological differences between organs. For Reinert et al. [15], ROIs were drawn only in lymph nodes, and in other studies, often only one organ is chosen [9,10,11,12]. Some exceptions exist in the literature when the pathology is disseminated to many organs (metastasis) [8].

Furthermore, all examinations were not performed on the same CT scanner. The technical differences may have increased the variability of lesion attenuation and affected the texture analysis results [18]. However, this is frequently the case in clinical practice; CT scans are often done in semi-emergency situations because of predominant chest involvement in PHL, and this pattern is also reported in the literature [10,15]. To evaluate the influence of inter-scanner differences on texture analysis, Choi et al. [10] compared the texture parameters of different CT scanners. They found that except for two parameters (gray-level co-occurrence matrices inverse difference moment and sphericity), no significant difference existed in texture analysis. We did not use the two parameters influenced by technical differences.

## Conclusion

No textural parameters were identified as predictors of ERA after two cycles of chemotherapy in PHL. A study that includes a larger population and selects texture parameters more accurately would be interesting.
